# Complete Genome Sequence of Ornithobacterium hominis Type Strain MSHR-COH1 (ATCC TSD-185)

**DOI:** 10.1128/mra.00379-23

**Published:** 2023-06-29

**Authors:** Susannah J. Salter, Robyn L. Marsh, Julian Parkhill

**Affiliations:** a Department of Veterinary Medicine, University of Cambridge, Cambridge, United Kingdom; b Menzies School of Health Research, Charles Darwin University, Darwin, Australia; c School of Health Sciences, University of Tasmania, Hobart, Australia; The University of Arizona

## Abstract

We report the complete genome sequence of the Ornithobacterium hominis type strain MSHR-COH1 (ATCC TSD-185/NCTC 14317), a bacterial species isolated from the human nasopharynx. Long-read sequencing reveals that the genome is 2,036,909 bp in length, with a GC content of 35.72%.

## ANNOUNCEMENT

Ornithobacterium hominis is a species in the family *Weeksellaceae*, for which partial genomes were first described from nasopharyngeal samples from Thailand (1). The first cultured isolates were recovered from Australia (2), including type strain MSHR-COH1 from a nasopharyngeal swab sample collected in 2009 from an infant with chronic suppurative lung disease (3).

Previous assemblies (1, 2) typically featured contigs bounded by nearly identical 450-bp noncoding sequences; short-read data alone were not adequate for generation of a closed assembly. To aid O. hominis comparative genomics, we have produced a circular genome of the type strain using long-read data polished with short reads.

O. hominis was acquired from the American Type Culture Collection (ATCC) (ATCC TSD-185), streaked on Columbia agar with 5% sheep blood (product number PB0123A; Thermo Fisher Scientific), and incubated at 37°C for 72 h under humid microaerobic conditions (>90% relative humidity, with 8 to 9% O_2_ and 7 to 8% CO_2_) using the CampyGen atmosphere generation system (product number CN0025; Oxoid) plus approximately 20 mL water. All bacterial colonies were scraped into phosphate-buffered saline and immediately processed using the MasterPure complete DNA/RNA purification kit (product number MC85200; BioSearch Technologies) following the manufacturer’s protocol. The sequencing library was prepared with the 24-plex native barcoding kit v12 (product number SQK-NBD112.24; Oxford Nanopore Technologies [ONT]). No DNA shearing or size selection was employed.

The library was sequenced on an ONT MinION device with an R10.4 flow cell (product number FLO-MIN112; ONT), with Guppy v6.4.6 base calling using the SUP model and adaptor/barcode removal. Reads were trimmed using Prowler (4) to a Q20 average in a 1,000-nucleotide window, discarding reads of <5 kb. The final data set contained 19,587 reads, with an *N*_50_ value of 10,303 bp and an *N*_90_ value of 5,608 bp.

Assembly was undertaken with default settings unless otherwise indicated. Twelve preliminary assemblies were generated with Flye v2.9.2 (5) using 64× overlapping subsamples of the reads, from which a circularized consensus was prepared with Trycycler v0.5.3 (6). The genome was rotated to the putative origin of replication, as inferred by Ori-Finder 2022 (7), and then polished with Pilon v1.24 (8) using previously published data (Illumina NextSeq 500 paired-end 150-bp reads [GenBank accession number SRR8360295]) mapped with Minimap2 v2.24 (9) to an average depth of 600×. This polishing step corrected 19 nucleotide errors and 28 indels. Finally, the genome was annotated with the Prokaryotic Genome Annotation Pipeline (PGAP) v6.5 (10).

**FIG 1:**
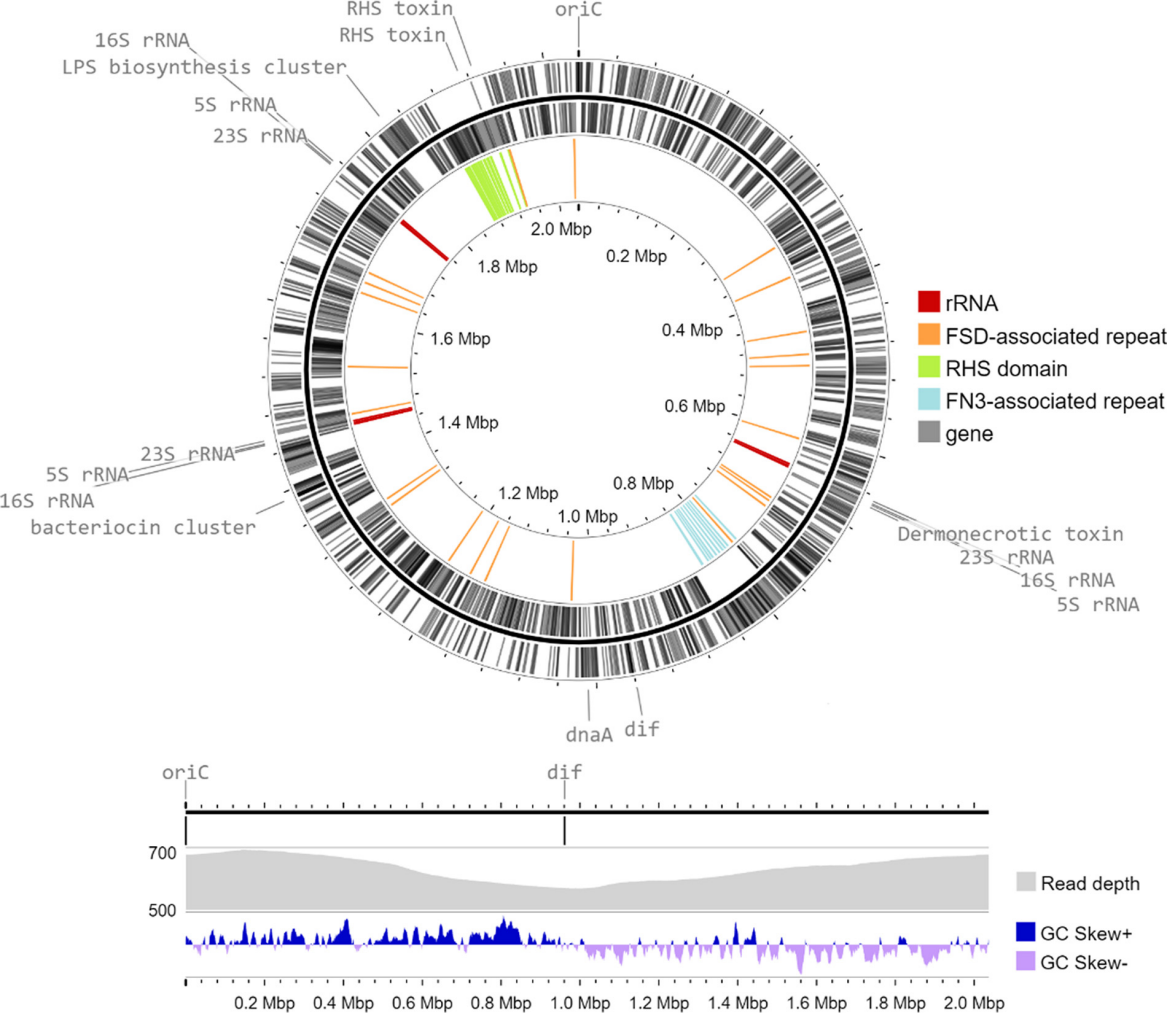
(Top) Circular chromosome of O. hominis MSHR-COH1, with genes in gray and the locations of repeats that may disrupt short-read assemblies on the inside ring. Landmarks around the genome are labeled, including the putative dermonecrotic toxin gene, a bacteriocin cluster, a lipopolysaccharide (LPS) biosynthesis cluster, and rRNA gene loci. (Bottom) Proposed origin of replication and *dif* sites, concordant with GC skew and mapped read depth.

Figure 1 was generated using Proksee (11) with the integrated CGView Builder v1.1.2 and GC Skew v1.0.1 tools. Read depth was calculated from the Minimap2 short-read output with a 500-kb sliding window.

The genome of O. hominis MSHR-COH1 consists of a single circular chromosome of 2,036,909 bp, with a GC content of 35.72%, 1,899 genes (of which 1,830 are coding sequences), and 3 rRNA operons. Sites that may disrupt short-read assemblies include the rRNA operons, 450-bp noncoding sequences associated with putative Fibrobacter succinogenes domain genes, >500-bp rearrangement hotspot (RHS) domain sequences, and >1-kb sequences within putative type III fibronectin domain genes. Unusually, the *dnaA* gene is situated near the proposed genome terminus rather than near the origin of replication (Fig. 1). The putative *dif* sequence, similar to that of Escherichia coli, and the proposed origin are both concordant with the positive GC skew on the leading strand, the greater mapped read depth near the origin of replication, and the directionality of rRNA operons.

The following software was used: Guppy v6.4.6 (ONT), Prowler (commit ID c3041ba) (https://github.com/ProwlerForNanopore/ProwlerTrimmer), Flye v2.9.2 (https://github.com/fenderglass/Flye), Trycycler v0.5.3 (https://github.com/rrwick/Trycycler), Ori-Finder 2022 (http://tubic.tju.edu.cn/Ori-Finder2022/public/index.php) (accessed 11 January 2023), Pilon v1.24 (https://github.com/broadinstitute/pilon), Minimap2 v2.24 (https://github.com/lh3/minimap2), and Proksee (https://proksee.ca) (accessed 27 April 2023).

### Data availability.

Data are available from the European Nucleotide Archive (ENA) under BioProject accession number PRJEB61795, run accession number ERR11281217, and assembly accession number GCA_951229915.1.
